# Gene cloning and molecular characterization of a thermostable chitosanase from *Bacillus cereus* TY24

**DOI:** 10.1186/s12896-022-00762-6

**Published:** 2022-10-27

**Authors:** Rong-Xian Zhang, Zhong-Wei Wu, Shu-Juan Zhang, Hui-Min Wei, Cheng-Wei Hua, Lan Li, Tian-You Yang

**Affiliations:** School of Life Science and Technology, Henan Institute of Science and Technology, Xinxiang, 453003 People’s Republic of China

**Keywords:** *Bacillus cereus*, Chitosanase, *choe*, Recombinant expression, Thermostability

## Abstract

**Background:**

An important conceptual advance in health and the environment has been recognized that enzymes play a key role in the green processing industries. Of particular interest, chitosanase is beneficial for recycling the chitosan resource and producing chitosan oligosaccharides. Also, chitosan gene expression and molecular characterization will promote understanding of the biological function of bacterial chitosanase as well as explore chitosanase for utilizing chitosan resources.

**Results:**

A chitosanase-producing bacterium TY24 was isolated and identified as *Bacillus cereus*. Moreover, the chitosanase gene was cloned and expressed in *Escherichia coli*. Sequence analysis reveals that the recombinant chitosanase (CHOE) belongs to the glycoside hydrolases 8 family. The purified CHOE has a molecular weight of about 48 kDa and the specific activity of 1150 U/mg. The optimal pH and temperature of CHOE were 5.5 and 65 °C, respectively. The enzyme was observed stable at the pH range of 4.5–7.5 and the temperature range of 30–65 °C. Especially, the half-life of CHOE at 65 °C was 161 min. Additionally, the activity of CHOE was remarkably enhanced in the presence of Mn^2+^, Cu^2+^, Mg^2+^ and K^+^, beside Ca^2+^ at 5 mM. Especially, the activity of CHOE was enhanced to more than 120% in the presence of 1% of various surfactants. CHOE exhibited the highest substrate specificity toward colloid chitosan.

**Conclusion:**

A bacterial chitosanase was cloned from *B. cereus* and successfully expressed in *E. coli* (BL21) DE3. The recombinant enzyme displayed good stability under acid pH and high-temperature conditions.

**Supplementary Information:**

The online version contains supplementary material available at 10.1186/s12896-022-00762-6.

## Introduction

Chitosan is a natural linear polymer, composed of a major constituent of D-glucosamine (GlcN) and randomly incorporated of N-acetyl glucosamine (GlcNAc) with β-1,4 bonds, exhibiting stable structure, which results in its poor solubility at neutral pH condition. Therefore, though chitosan has potential uses as functional material because of its attractive properties such as biocompatibility and biodegradability, the poor solubility limits its further application [1]. Whereas, the hydrolysis products of chitosan, biological active COSs (chitosan oligosaccharides), with solubility in water and absorbability by the body, have potential in a wide range of applications, such as agriculture, health food, cosmetics and biomedical fields [2, 3].

Conventionally, the preparation methods of COSs include chemical method that involves detrimental chemicals such as acid, H_2_O_2_, and NaNO_2_, and physical method. The processes usually require high energy consumption, and severe reaction conditions, resulting in uneven products, toxins to animals as well as discharge of pollutants into the environment. Alternatively, there have been reports on enzymatic degradation of chitosan to produce COSs in the past few decades [2]. Enzymatic hydrolysis of chitosan generally proposes preferable to physical and chemical methods with mild reaction conditions, environmental safety, and greater selectivity. Chitosanase (EC. 3.2.1.132) is a group of enzymes, hydrolyzing β-1,4-glucosidic bonds in chitosan to produce COSs, which has drawn substantial attention from researchers. Recently, *Paenibacillus barengoltzii* chitosanase, *Bacillus amyloliquefacien*s chitosanase, *Bacillus* sp. chitosanase, and *Asperillus griseoaurantiacus *chitosanase have been reported for preparation of COSs [4–7]. Also, it has been reported that a variety of microbial chitosanases are produced by bacteria, particularly the genus of *Bacillus*, such as *B. amyloliquefaciens* DSMZ, *Bacillus cereus* S1, *Bacillus* sp. KCTC 0377BP and BY01, and *Bacillus subtilis* SH21 [5, 8–11]; fungi, such as *Aspergillus fumigatus* ATCC13073 and *Penicillium* sp. D-1 [12, 13]; actinomycetes, such as *Streptomyces* sp. [14]. However, to a certain degree, the catalytic potential in the degradation of chitosan, chitosanase still currently can’t meet the industrial requirements of industrial production because of its limited efficiency and sustainability. Therefore, exploring novel chitosanase resources and gene expression is still of great importance to utilize the chitosan resource.

*Escherichia coli* is usually used as the expression system to achieve high expression of protein including chitosanases because of its high production efficiency, shorter fermentation period, and low-cost medium. For example, the chitosanase-encoding genes from various microorganisms, such as *Bacillus* sp. BY01 and TS, *B. subtilis* V26, *Gynuella sunshinyii*, *Streptomyces avermitilis* and *Penicillium* sp. D-1, were successfully expressed in the *E. coli* system [10, 13, 15–18]. While the formation of the recombinant chitosanase from *A. Fumigatus* in *E. coli* BL21 (DE3) was inclusion body [19]. It is also reported that the chitosanase gene (CsnQ) from *Bacillus* sp. Q1098 has been cloned and heterologously expressed, which exhibited more than 50% of activity over pH stability [6].

In this work, a chitosanase-producing *B. cereus* TY24 has been isolated and identified from shrimp and crab compost samples. Moreover, the chitosanase-encoding gene (*choe*) from the isolate was cloned and successfully expressed in *E. coli*. Furtherly, the molecular characterization of CHOE was studied.

## Materials and methods

### Materials

*E. coli* JM109 and *E. coli* BL21 (DE3) were used for cloning of 16 S rDNA fragment and target chitosanase gene (*choe*), and expressing *choe*, respectively. The plasmid pET 28a(+) was used for expression. Takara Bio Inc (China) supplied such as pMD 19-T vector, T4 DNA ligase, *Bam*H I, *Hin*d III, and Extaq. Beyotime Biotechnology (China) offered Bradford protein assayed kit. Chitosan was supplied by Sangon Biotech (China).

### Isolating and screening of chitosanase microbial producer

The strains were isolated from shrimp and crab shell compost samples in Qingdao city, Shangdong province. The primary isolating medium was composed of (g/l): colloid chitosan 5, MgSO_4_ 1.4, K_2_HPO_4_ 4, KH_2_PO_4_ 2, NaCl 1, KCl 1, CaCl_2_ 0.2, Yeast Extract 1, pH 7.0. The secondary isolating medium was composed of (g/l): colloid chitosan 5, K_2_HPO_4_ 1.4, KH_2_PO_4_ 0.6, MgSO_4_ 1.0, Yeast Extract 0.6, Peptone 20, pH 7.0. Strains with clear zones on the chitosan agar were selected for further study. And the chitosanase activities were detected in culture in the chitosan medium.

### Strain identification

Morphological observation. The properties of the bacterial colonies were observed after cultivation in the LB agar plate and Gram stain. The bacterial cells at logarithmic phase in LB medium were collected by centrifugation (998 *× g*, 2 min) and washed with PBS buffer. Having been coated and fixed on glass slides, the bacterial cells went through fixation with 2.5% glutaraldehyde for 3 h, dehydration with gradient ethanol at the concentrations of 30%, 50%, 70%, 100% and 100%, then spray gold treatment with Sputter Coater (Hitachi E-1010). The bacterial cells were observed with Quanta 200 scanning electron microscope (SEM, FEI company, USA), and the images of the bacterial microscope were taken.

Molecular identification. The bacterial genome of Strain TY24 was used as the template. The 16 S rDNA sequences were amplified by polymerase chain reaction (PCR) with the pair of P0 and P6 primers (P_0_ primer: GAGAGTTTGATCCTGGCTCAG; P_6_ primer: CTACGGCTACCTTGTTACGA). The PCR proceeded: firstly, 95 °C for 3 min; secondly, 30 cycles of denaturing at 94 °C for 15 s, annealing at 56 °C for 15 s, and extending at 72 °C for 90 s; finally, extension at 72 °C for 10 min. The ligation product of the PCR product and the cloning vector (pMD19 T) was transferred into the cloning host, resulting in *E. coli* JM109 with the vector which was incubated in LB medium with 100 µg/ml ampicillin at 37 °C. The positive transformants were verified by PCR method, then the target DNA fragment was sequenced by GeneCreate Biological Engineering Co., Ltd. (Wuhan, China). After the blast of the 16 S rDNA sequences in National Center of Biotechnology Information (NCBI) database, the phylogenetic tree based on their similarities was constructed using the neighbor-joining method by the MEGA 7 software.

### Preparation of colloidal chitosan

According to the methods described by Shehata et al. and Kurakake et al. [7, 8], the 1% colloidal chitosan was prepared as follows: one gram of chitosan was dissolved in about 80 ml the sodium acetate buffer (100 mM, pH 5.5) and continuously stirred about 2 h. After the chitosan was completely dissolved, the solution was adjusted to different pH values by 1.0 M sodium acetate or sodium hydroxide, and finally made up to 100 ml by adding the sodium acetate buffer.

### Chitosanase activity

The 3,5-dinitrosalicylic acid (DNS) method was used to determine the chitosanase activity [20]. The reaction system was composed of 0.9 ml 1% substrate, 0.1 ml enzyme solution and 1 ml sodium acetate buffer (pH 5.5, 50 mM). After the mixture has been inoculated at 55 °C for 20 min, the reaction was terminated by boiling for 10 min. Then, the chromogenic reaction was performed by adding 1.5 ml DNS solution, and kept at 100 °C for 5 min. The control was conducted by using the deactivated enzyme solution under the identical condition. Furtherly, the content of reducing sugar was measured by the absorbance at the wavelength of 540 nm (A_540_), using D-glucosamine as the standard [15]. One unit of chitosanase activity was defined as the amount of enzyme quantity for producing 1 µmol of reducing sugar per minute under the conditions described above.

### Sequence analysis of *choe* and ChOE, and homology modeling of CHOE

#### Cloning of *choe* gene

Based on the relationship of *B. cereus* producing chitosanases, it is deduced that *B. cereus* TY24 secreted chitosanase. The primers (*choe*-F and *choe*-R) were designed according to the reported chitosanase-encoding gene from *B. cereus* A8 with the accession number (WP_139019914.1) of the family glycosyl hydrolases 8 (GH-8) (choe primer: CGGGATCCATGAATGGAAAAA (BamH I); choe primer: CCCAAGCTTTTATTATCGTA (Hind III). The chitosanase encoding chitosanase gene (*choe*) was amplified by PCR using the genomic DNA of the isolate TY24 as the template and the pair of primers (*choe*-F and *choe*-R). The purified restricted product and the vector pET-28a(+) were digested by the double restriction endonucleases digestion of *Bam*H I and *Hin*d III, and subsequent ligation resulted in the pET-28a(+)-*choe* plasmids. The generated vectors were transformed into the cloning host of *E. coli* JM109 and the expressing host of *E. coli* BL21(DE3) in sequence. Meanwhile, the positive transformants were selected with 50 µg/ml kanamycin and verified by the *Bam*H I -*Hin*d III digestion as well as PCR method with the general primers (T7 + and T7 −) of pET 28a(+) vector. Furtherly, the target gene was sequenced by GeneCreate Biological Engineering Co., Ltd.

#### Protein structure analysis of CHOE

The amino acid sequence of CHOE. After the blast in the NCBI database, the homology analysis of deduced protein sequences was performed by using DNAMAN software with related sequences retrieved from the NCBI database. Signal peptide of CHOE was analyzed by SignalP-5.0 prediction Server (https://services.healthtech.dtu.dk/service.php?SignalP-5.0).

Homology modeling. The secondary structure of CHOE was with recognition and alignment with the structure of the chitosanase from *Bacillus* sp. K17 (PDB number: 1v5d) by Phyre2 (http://www.sbg.bio.ic.ac.uk/phyre2) [21, 22]. The three-dimensional (3D) homology model of CHOE was built by SWISS MODEL (https://swissmodel.expasy.org). The crystal structure of chitosanase with PDB number 7cju in the PDB database (https://www.rcsb.org/) was used as the template [23]. The quality of structure was evaluated and verified by structure analysis and verification server with the ERRAR and VIFIFY 3D and PROCHECK.

### Heterologous expression and production of the recombinant chitosanase (CHOE)

*E. coli* BL21 (DE3)/the pET-28a(+)-*choe* plasmids were cultivated in Terrific Broth (TB) medium with Kan and supplemented with isopropyl-β-D-thiogalactopyranoside (IPTG) for induction and antibiotics (50 µg/ml kanamycin). Induction was performed at the lower temperatures and 200 rpm for several hours, when absorbance at the wavelength at 600 (A_600_ nm) of the culture reached about 0.7, after being cultured at 37 °C and 200 rpm. The production of recombinant chitosanase was optimized via inducting conditions by adding IPTG at 0.025 mM to 0.75 mM, at temperatures from 16 to 37 °C for several hours.

### Purification and SDS-PAGE analysis of CHOE

The recombinant cells were collected by centrifugation (4746 × *g*, 10 min and 4 °C), dissolved in the phosphate buffer (50 mM NaH_2_PO_4_–Na_2_HPO_4_, 500 mM NaCl), and then subjected to ultrasonic crush (3 s on and 5 s off) for 30 min. After centrifugation and filtration by the 0.22 μm filters, the supernatant was collected as the crude enzyme solutions. The chitosanase was purified by Ni-NTA affinity column (HisTrap™ FF, GE Healthcare Life Sciences China). The target protein was purified with the different imidazole concentrations in the buffer. The purified enzyme was kept at 4 °C for further analysis.

After determination of protein content by the Bradford method [24], samples were pretreated with the SDS loading buffer by boiling for 5 min, then were analyzed by SDS-PAGE with 10% (w/v separating polyacrylamide gel) according to the Laemmli [25]. The gels were stained with Coomassie brilliant blue R-250.

### Characterization of CHOE

#### Effects of pH and temperature on CHOE activity

The optimal pH and temperature of recombinant chitosanase were determined in the pH range of 4.0–7.5 (sodium acetate buffer, pH 4.0–6.0; sodium phosphate buffer, pH 6.0–7.5) and temperature range of 30–75 °C at 5 °C intervals. Moreover, the pH stability and thermostability of CHOE were determined. The initial activity and the remaining activity were determined before and after being incubated for 1 h under the corresponding conditions. All tests were carried out in triplicate. Moreover, the half-life (t_1/2_) of CHOE at 65 °C was determined and calculated according to the formula of ${\text{t}}_{1/2}=\text{ln}2/k$. In the formula, *k* represented the slope of logarithmic residual chitosanase activity. The residual chitosanase activities were tested at 20 min intervals.

#### Effects of chemicals on the chitosanase activity

Chemicals including metal ions, Ethylene Diamine Tetraacetic Acid (EDTA) and surfactants were used to test the effects on chitosanase activity. The purified CHOE was preincubated with chemical reagents for 30 min at room temperature. The activities were assayed at the optimal pH and temperature.

#### Substrate specificity of CHOE

To test the preference of CHOE toward different substrates, the substrate specificity was assayed at the optimal pH and temperature conditions. The substrates including colloidal chitosan, chitosan powder, sodium carboxymethylcellulose and carboxymethyl chitosan were used at the concentration of 1%.

#### Kinetic parameters of CHOE

The kinetic parameters of the purified recombinant chitosanase were performed with colloid chitosan substrate at different concentrations (0.1–1.5%) at the optimal pH and temperature conditions. The kinetic parameters, the maximal catalytic rate (*V*_max_) and Michaelis-Menten constant (*K*_m_) were calculated according to Lineweaver-Burk using GraphPad Prism version 8.0.

### Data analysis

Values are presented as the means of triplicates ± standard deviation.

## Results and discussion

### Screening, isolation, and identification of microbial chitosanase producers

**Fig. 1:**
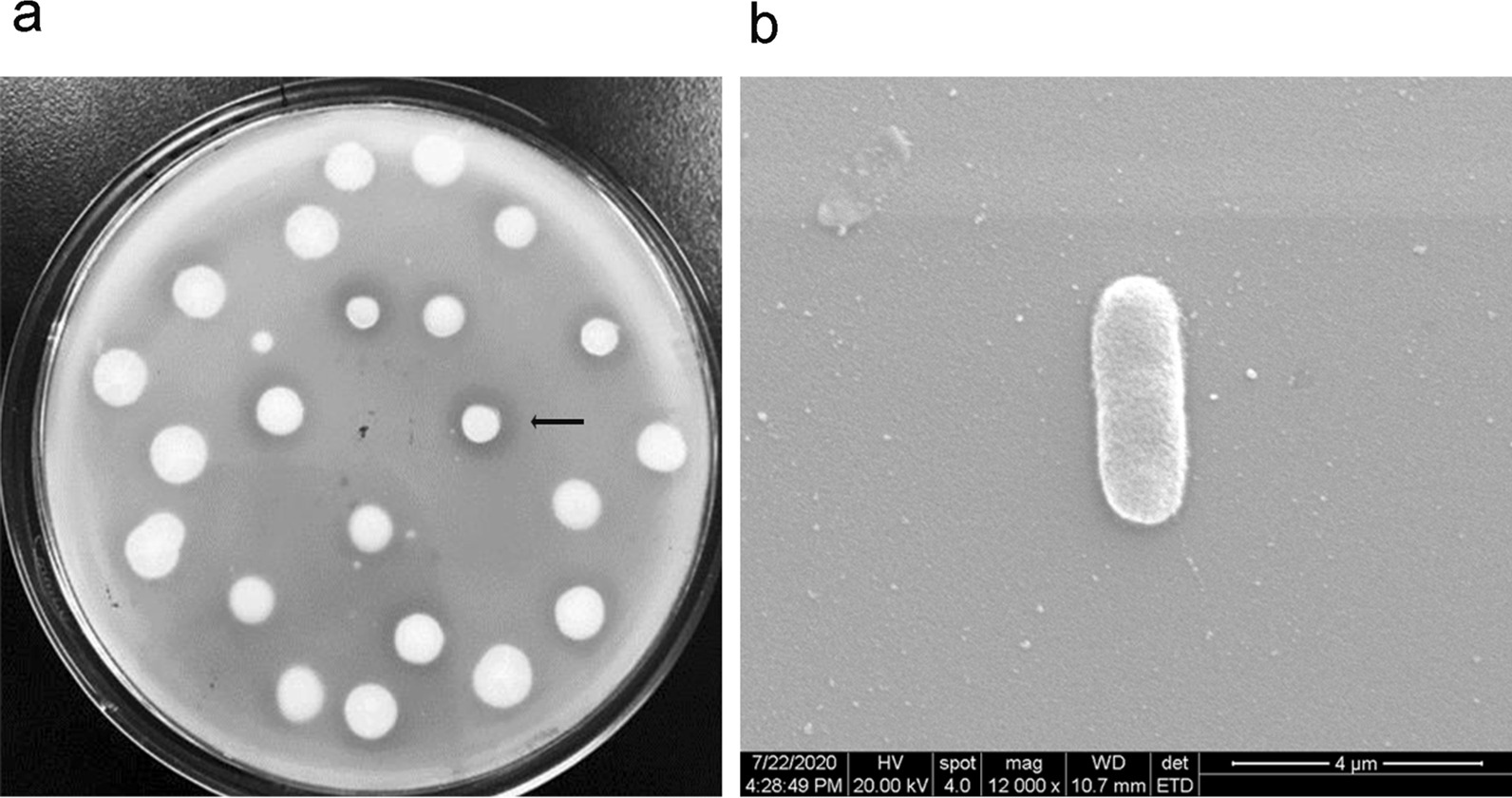
Microbial morphologies of the isolate TY24. **a** The strains on a colloid chitosan agar plate, TY24 was indicated by the arrow; **b** The image of TY24 observed by SEM

In this study, the chitosanase-producing strains were screened and isolated using chitosan as the main carbon source from the isolated chitosan and chitin-enriched samples. Thirty-seven microbial strains were newly isolated from the samples and identified as chitosanase producers based on their zone formation patterns on chitosan-containing media (Fig. 1a). The most active positive colonies with clear zones were selected for further study. Six isolates showed high chitosanase activity among them. Strain TY24 showed the highest extracellular chitosanase activity (4 U/ml), and accordingly, was selected for further study.

**Fig. 2:**
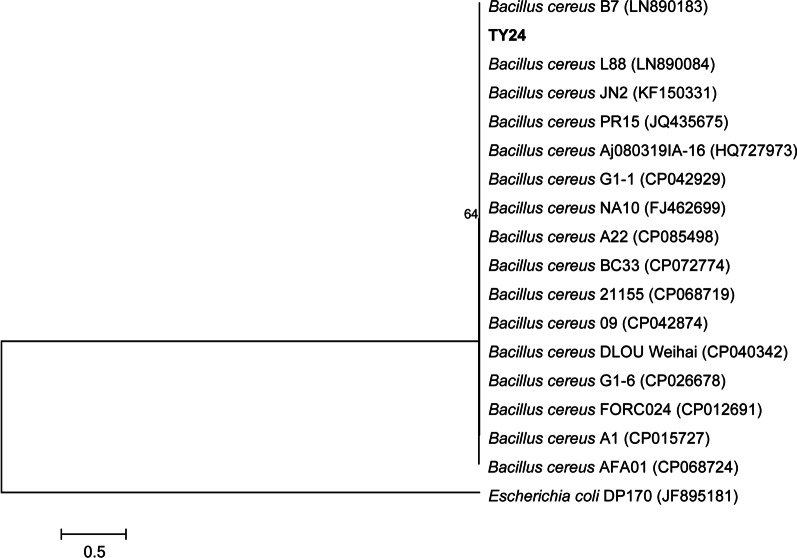
Phylogenetic tree of the strain TY24 based on the 16 S rDNA sequences with the neighbor-joining method. The scale bar indicates the average number of substitutions per site. The bootstrap test of the tree was performed with 1000 replicates. The *E. coli* DP170’s 16 S rDNA sequence was used as the outgroup

The morphology of colonies of YT24 was a little yellow-white in color, and rough on the surface and at the edge on LB agar medium plate. The micromorphology of the strain was Gram-positive. The cell is spore-forming and rod in shape (Fig. 1b). The results suggest it belongs to the genus of *Bacillus*. The molecular identification of the strain was also further performed. The isolate’s 16 S rDNA sequence that was submitted to the GenBank database, obtained the accession number of ON506254. Based on the blast and alignment of the partial 16 S rDNA sequence of TY24 with the related sequences gathered from the NCBI database, the constructed phylogenetic tree showed that Strain TY24 has 99% similarity to *B. cereus* strains (LN890183, JQ435675, and CP042929), which suggested that the isolate TY24 located in the clade of *B. cereus* (Fig. 2). Because of high growth rates and the extracellular protein products, *Bacillus* species including *Bacillus* sp., *B. subtilis* and *B. licheniformis*, are reported as industrial microorganisms.

### Cloning and sequence analysis of *choe* and CHOE

**Fig. 3:**
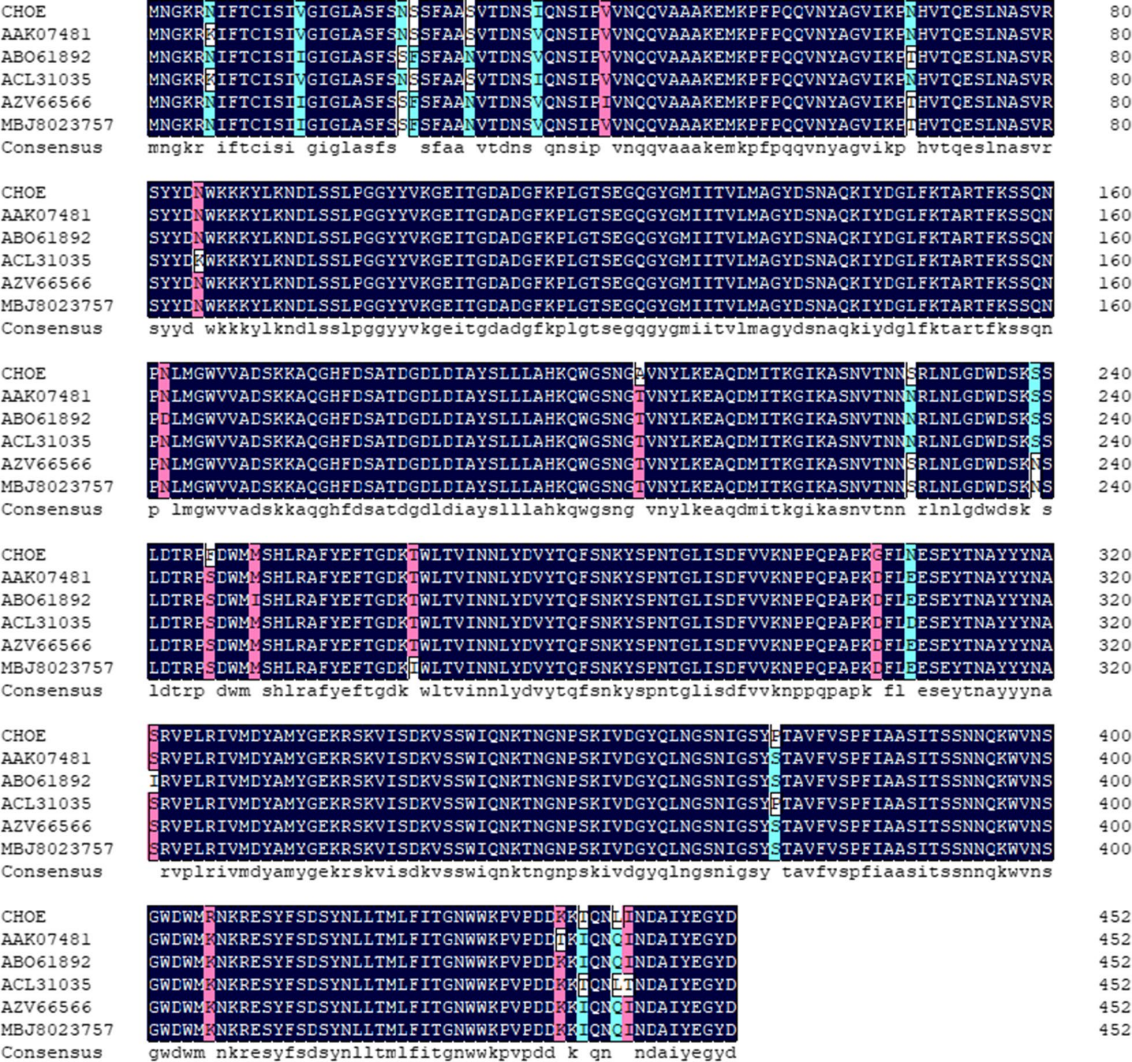
Sequence comparison of CHOE and other chitosanases from the family GH-8. CHOE, *B. cereus* TY24; AAK07481, *Bacillus* sp. KCTC 0377BP; ABO61892, *Bacillus thuringiensis* serovar israelensis; ACL31305, *Bacillus* sp. S-1; AZV66566, *B. cereus* ZB201708; MBJ8023757, *B. cereus* N26

The chitosanase-encoding gene (*choe*) cloned into the pMD 19T vector was sequenced. The gene contains a 1362-bp ORF in length, which results in a chitosanase (CHOE) of the 453-amino acid residues protein chain. The analysis of CHOE by the SignalP-5.0 prediction Server shows that CHOE contains a 27-aa signal peptide in its N-terminus. Based on the sequence alignments of the NCBI Database, CHOE exhibited 96% of identity with the chitosanases from *B. thuringiensis* serovar israelensis (ABO61892), *Bacillus* sp. KCTC 0377BP (AAK07481), *Bacillus* sp. S-1 (ACL31305), *Bacillus cereus* ZB201708 (AZV66566), *Bacillus cereus* N26 (MBJ8023757) (Fig. 3). Also, based on the CDD (the conserved domain database) [26], the amino acid residues of Asp-122 and Glu-183, and the amino acid sequence of ATDGDLDIAYSLLLAHKQWGSNG were found highly conserved as the catalytic residues, and proved as the typical conserved region of the family GH-8, respectively. Therefore, CHOE was presumed to belong to the family GH-8.

**Fig. 4:**
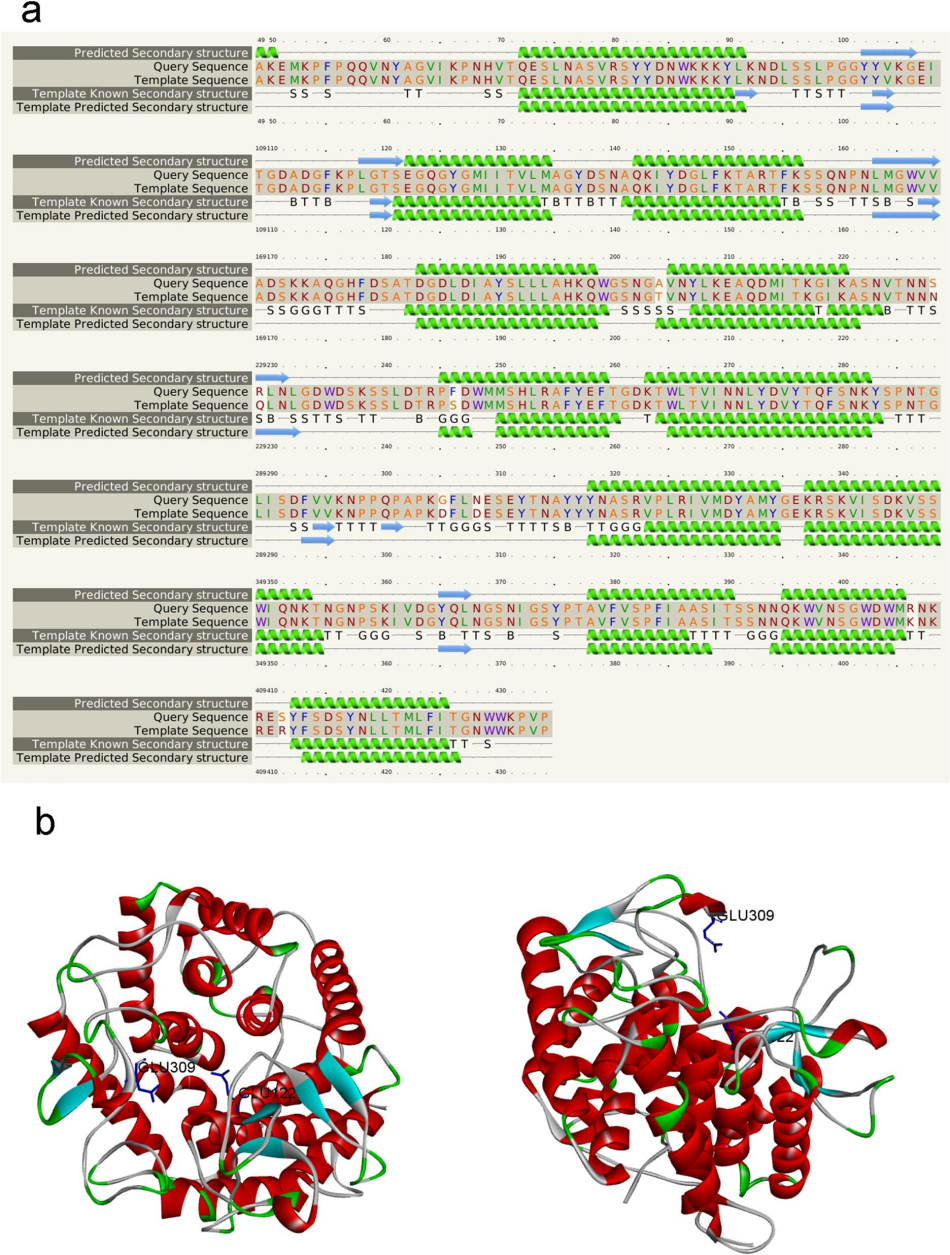
Bioinformatic analysis of CHOE structure. **a** Secondary structure of CHOE analyzed by Phyre2 server; **b** Homology model of CHOE in a top view (left) and a side view (right), the catalytic sites, Glu122 and Glu309, were shown with blue sticks

The secondary structure of CHOE was aligned with that of the chitosanase from *Bacillus* sp. K17 (PDB number: 1v5d) by Phyre2 (98% identity) (Fig. 4a). The 3D model of CHOE was built by Swiss model server using the X-ray structure of the chitosanase from *Bacillus* sp. (PDB number: 7cju, 1.74 Å) as the template, which shares 97.69% similarity with CHOE from *B. cereus* TY24 (Fig. 4b) [23]. The quality of CHOE’s model was evaluated by Ramachandran plot, also verified by https://srv.mbi.ucla.edu/ server with ERRAR and VERIFY 3D functions. The analysis of the Ramachandran plot showed that 90.0%, 9.4% and 0.6% of total amino acid residues lay in the core zone, allowing region, and general region, respectively. The total reasonable region value of the Ramachandran plot is 99.4% (higher than 95%). Also, the value of ERRAT is 95.687% (higher than 85%), and the evaluation by VERIFY 3D was passed. In conclusion, the model of CHOE was reasonable. The overall 3D structure shows an α_6_/α_6_-double barrel in the structure, a typical feature of the family GH-8 [21], which suggests CHOE belongs to the family GH-8 (Fig. 4b).

### Expressing of *choe* and production of CHOE

**Fig. 5:**
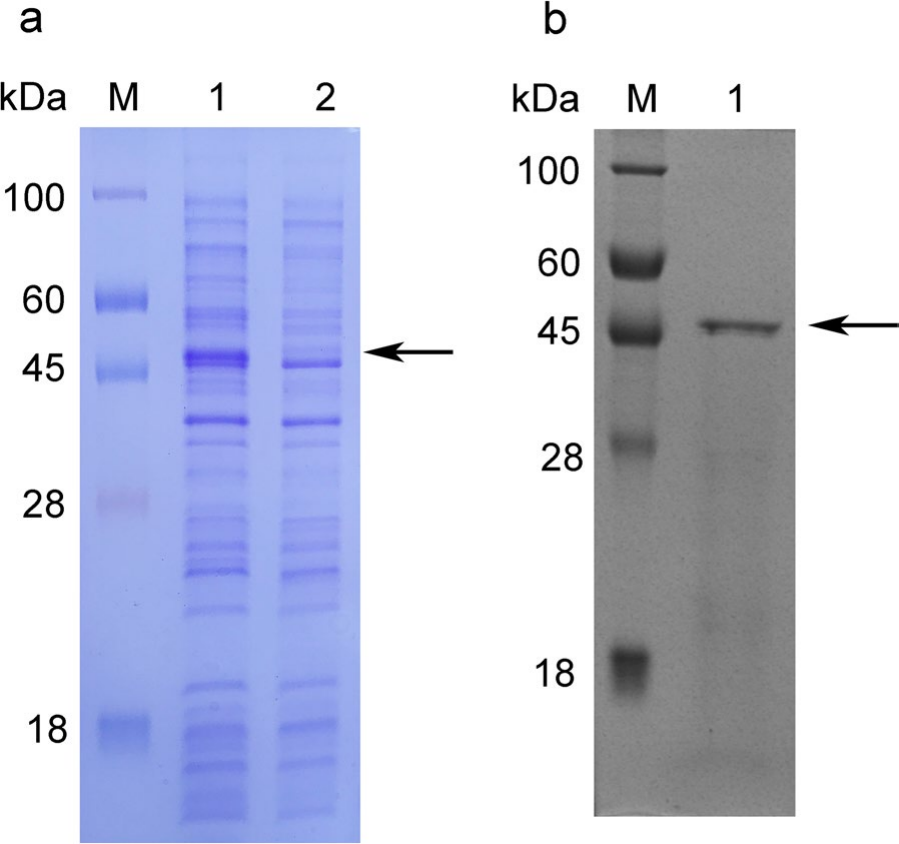
SDS-PAGE analysis of the recombinant chitosanase. **a** Lane M, protein standard molecular weight; lane 1, supernatant of *E. coli* BL21 (DE3)/pET28a(+)-*choe* cell lysate induced by IPTG; lane 2, supernatant of *E. coli* BL21 (DE3)/pET28a(+) cell lysate as control. **b** Lane M, protein standard molecular weight; lane 1, the purified chitosanase. The original gels are presented in Additional file 1: Figures S1 and S2, respectively

The *choe* gene was successfully expressed in *E. coli* BL(DE3) system (Fig. 5a). Chitosanase activity was detected in the resulting recombinant strain of *E. coli* BL21 (DE3)/pET 28a(+)-*choe* rather than in the control recombinant strain of *E. coli* BL21 (DE3)/pET 28a(+).

Since the recombinant chitosanase levels were affected by induction conditions, the effects of different expression temperatures, inducer concentrations, and induction time on the production of CHOE were determined in the study [27]. The CHOE production by the recombinant strains of *E. coli* BL (21) DE3/pET 28a(+)-*choe* were detected under the induction conditions, IPTG concentrations of 0.025–0.70 mM and temperature range of 16–37 °C. The results showed that when the recombinant strains were expressed at 25 °C, the chitosanase activity was higher than those induced at higher temperatures (30 and 37 °C) and low temperatures (16 and 20 °C). The reason might be that higher temperature enhanced the metabolism rate of cells, as well as accelerated the target protein’s synthesis, consequently, the proportion of the target protein’s active conformation was decreased; induction at lower temperatures caused the low growth rate of cells as well as the production of the target protein. In T7 promoter-based expression system, IPTG was usually used to induce the working of the expression system [27–30]. It was also observed that the chitosanase activity of CHOE reached the highest when IPTG was at 0.25 mM. Conclusively, the highest yield of the recombinant chitosanase was observed when the *E. coli* BL (21) DE3/pET 28a(+)-*choe* was induced by 0.25 mM IPTG at 25 °C for 9 h.

### Purification and SDS-PAGE analysis of CHOE

After induction, the recombinant cells were collected by centrifugation and washed with sodium acetate buffer (pH 5.5, 50 mM). The cells were broken by an ultrasonic crusher, and the supernatant was obtained by removing fragments of the cells by centrifugation, and further went through 0.22 μm membrane filtration. The recombinant enzyme was purified by Ni-NTA column. Meanwhile, SDS-PAGE analysis of the CHOE showed that the molecular weight of CHOE was approximately 48 kDa, consistent with its calculated molecular weight of 47.7 kDa (Fig. 5b). The molecular weight of CHOE was similar to the chitosanases of the family GH-8 produced by *Bacillus* sp. TS and *Bacillus* sp. KCTC 0377BP [9, 15]. The specific activity of the purified recombinant chitosanase was 1150 U/mg, which was higher than those of the chitosanases from various microorganisms, such as *Bacillus* sp. TS (555.3 U/mg) and *P. barengoltzii* (388.9 U/mg) [15, 31], and was similar to those of the chitosanase from *Kanthinobacterium* sp. 4239 (1500 U/mg) and BaCsn46A from *B. amyloliquefaciens* (1031.2 U/mg) [32, 33].

### Characterization of CHOE

#### Effects of pH on activity and stability of CHOE

**Fig. 6:**
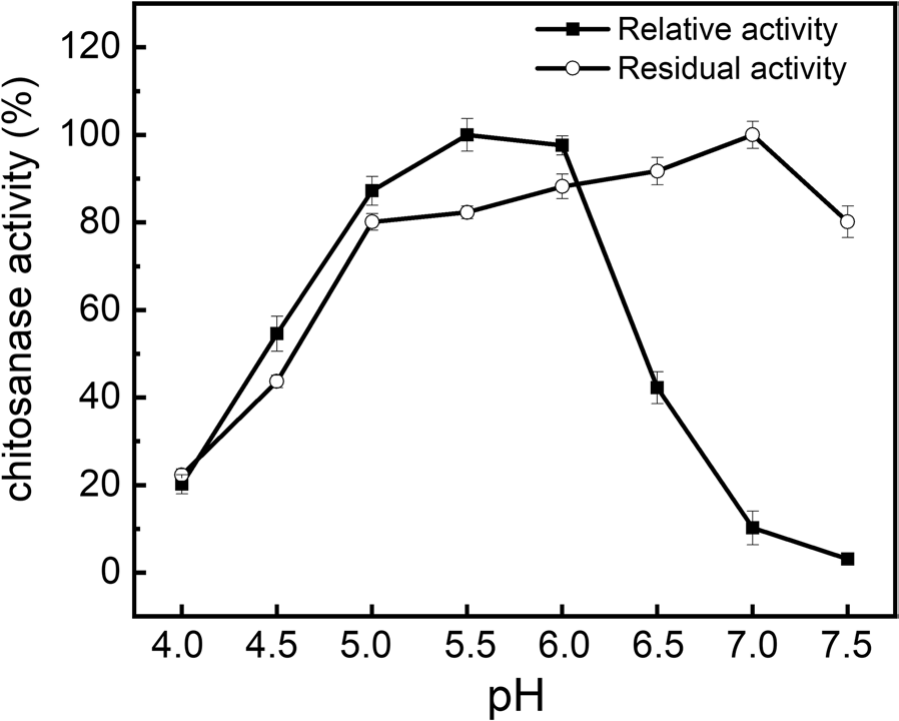
Effect of pH on the activity of the recombinant chitosanase

pH always affects not only the activity and stability of enzymes by means of protonating or deprotonating states of protein conformation, but also the conformation of chitosan substrate, for example, the better solubility of chitosan was detected in the acid environment. As shown in Fig. 6, the optimal pH of CHOE was determined to be 5.5, which is different from those of chitosanases from *B. cereus* (pH6.0) and *B. thuringiensis* (pH7.0) and *Aspergillus* spp. (pH6.0). Meanwhile, the relative activity was more than 80% and 54.58% of its maximum activity in the pH 5.0–6.0 and pH 4.5, respectively, suggesting that CHOE is an acidic chitosanase. Moreover, CHOE in an acidic environment was observed remarkably stable. As shown in Fig. 6, CHOE exhibited excellent stability in the pH range of 4.5–7.5, retaining more than 80% residual activity after pre-incubating for 1 h. Because of the low solubility of chitosan when the environmental pH value is higher than 6.2, chitosanases with optimal pH and stability in the acid environment is particularly popular in commercial and industrial application. The results indicated that CHOE has a good adaption in the acid environment. It is deduced that though the chitosanases from *B. cereus* TY24, *Bacillus* sp. TS, *B. cereus* S1 and *B. thuringiensis* have high sequence similarity, they exhibited distinctive catalytic characteristics [8, 15, 34].

#### Effects of temperature on activity and stability of CHOE

**Fig. 7:**
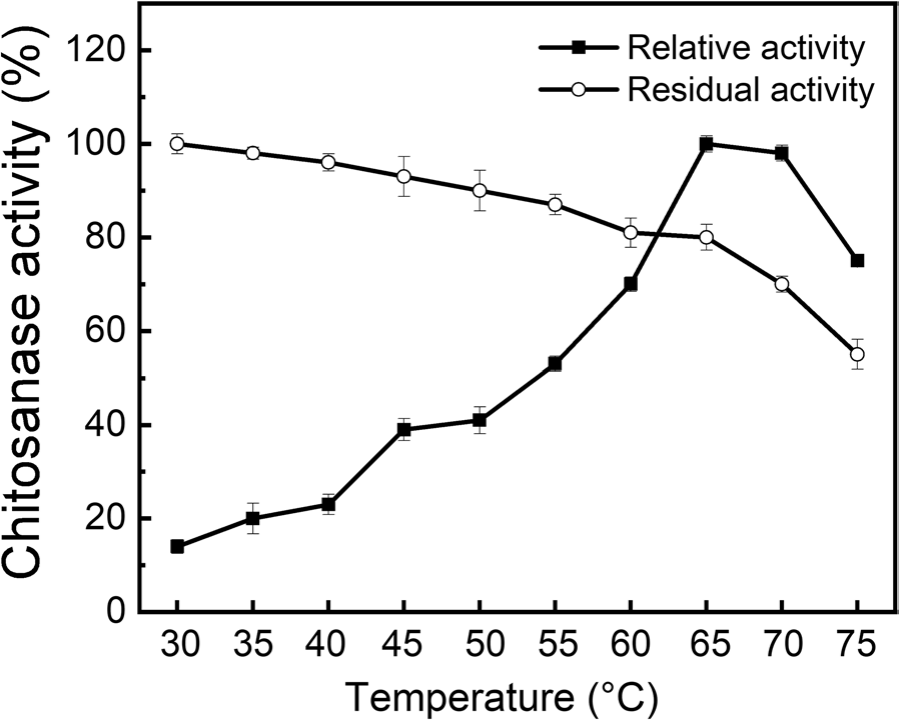
Temperature profile of the purified recombinant CHOE

As shown in Fig. 7, CHOE showed the optimal temperature of 65 °C and relatively high activity in the temperature range of 60–75 °C (more than 70%). The optimal temperature of CHOE was higher than the chitosanase from *Bacillus* sp. TS. (60 °C), Csn-PD from *P. dendririoformis* and the chitosanase II from *A. fumigatus* ATCC 13,073 (40 °C) [4, 12, 15]. Interestingly, CHOE performed remarkably thermo-stability in the range of 30–70 °C. The residual activity was more than 80% at 30–65 °C, 70% at 70 °C, and unstable at above 75 °C (Fig. 7). It has been reported that the engineered CsnA from *Renibacterium* sp. QD1 for improving thermostability and the chitosanase from *Bacillus* sp. S65 retained about 40% activity and less than 10% after being kept at 60 °C for 60 min and at 65 °C for 10 min, respectively [35, 36]. Also, the engineered chitosanase from *Bacillus* sp. TS. Moreover, the t_1/2_ of CHOE was determined to be 161 min at 65 °C, which is superior to the variant chitosanase from *Bacillus* sp. TS with increasing thermostability with a t_1/2_ of 35 min at 60 °C [37]. It is known that excellent thermostability of enzymes is beneficial for accelerating bioconversion efficiency, and consequently decrease of the cost during the enzyme’s application because the good thermostability of chitosanase can allow to improve reacting rate, decrease the viscosity of substrate, also minimize the risk of microbial contamination. Consequently, CHOE with notably attractive thermostability indicates its potential application.

#### Effects of chemicals on activity of CHOE

The effects of various chemicals, including metal ions, EDTA and surfactants on the chitosanase activity were determined (Table 1). Among the tested metal ions, K^+^, Ca^2+^, Mn^2+^, Mg^2+^, and Cu^2+^ exhibited obvious activating effects on the chitosanase activity of CHOE, Wherein, Ca^2+^ enhanced the activity by 1.8-fold, which was in accordance with the chitosanases from *Bacillus* sp. MET 1299 and *Bacillus* sp. S65 [36, 38]. And Mg^2+^, K^+^ and Mn^2+^ showed obviously stimulatory effects on the activity of CHOE (about the increase of 1-fold). It has been reported that Mn^2+^ showed a stimulatory effect on the activities of chitosanases from *Bacillus* sp. MET 1299 and *Pseudomonas* sp. OUC1 [38, 39], but the inhibitory effect on the chitosanase from *Bacillus* sp. strain KCTC 0377BP which also belongs to the family GH-8 [9]. Interestingly, Cu^2+^ enhanced obviously the chitosanase activity to 153%. It has been reported that Cu^2+^ presented stimulatory effects on the activities of *Bacillus* sp. S65 chitosanase and *Bacillus* sp. BY01 chitosanase, and the inhibitory effect on chitosanases from *Aspergillus* sp. W-2 and *Penicillium* sp. D-1 in the previous reports [10, 13, 36, 40]. On the other hand, Zn^2+^ and Co^2+^ and Al^3+^ inhibited more than 50% of the chitosanase activity; the heavy metal ions, Hg^2+^, Pb^2+^ and Fe^3+^ completely inhibited the activity. The inhibitory effects of Co^2+^ and Hg^2+^ were also observed on the chitosanases from *Bacillus* sp. TS and *Pseudomonas* sp. OUC1 [15, 39]; Fe^3+^ was reported as an inhibitor to the activity of *Penicillium* sp. D-1 chitosanase. Meanwhile, the activity of CHOE was complected inhibited in the presence of EDTA, the famous metal ion chelating agent, which indicated the metal ions played a key role in maintaining the activity of the enzyme. Attractively, it was observed that the activity of CHOE was notably activated by more than 20% in the presence of various surfactants, including Tween 20, Tween 80 and SDS at the concentration of 1% (Table 1).

**Table 1:** Effects of various chemicals on the CHOE activity

Metal ions	Concentrations	Relative activity (%)
K^+^	5 mM	198.08 ± 0.49
Ca^2+^	5 mM	260.16 ± 2.71
Zn^2+^	5 mM	47.84 ± 1.39
Mn^2+^	5 mM	180.67 ± 2.15
Fe^2+^	5 mM	23.38 ± 1.64
Mg^2+^	5 mM	217.53 ± 3.81
Cu^2+^	5 mM	153.21 ± 1.43
Co^2+^	5 mM	45.23 ± 2.18
Hg^2+^	5 mM	ND*
Pb^2+^	5 mM	ND
Fe^3+^	5 mM	ND
Al^3+^	5 mM	39.39 ± 1.28
EDTA	5 mM	ND
Tween 20	1%	135.15 ± 3.88
Tween 80	1%	120.32 ± 2.68
SDS	1%	122.15 ± 2.28

The sample with no addition of chemicals was defined as the control. ND means not detected

#### Substrate specificity of CHOE

The activities of CHOE towards various substrates were determined (Table 2). It was observed that the enzyme showed effective hydrolysis of colloidal chitosan (100%), subsequently, carboxymethylcellulose (8.21%). Additionally, CHOE showed low activity toward carboxymethyl chitosan (4.74%), and chitosan powder (3.34%). The results suggested that CHOE, the family GH-8 chitosanase, was capable of hydrolyzing the carboxymethylcellulose substrate. The reason might be the similar structural architecture of the (α/α)_6_-fold barrel to that of cellulase in the family GH-8 with the identical catalytic center and region which involve in hydrolyzing the β-1,4-linkage of the substrates [41]. Interestingly, it was observed that in comparison to chitosan powder and carboxymethyl chitosan, CHOE showed higher substrate activity toward carboxymethyl cellulose, which may be related to the conformation resulting from the space size and configuration of constitutional monomers, and the solubility of the different types of substrates. The substrate specificity of CHOE is accordant with the chitosanase (SaCsn46A) from *S. avermitilis*, which was also reported with high activity toward colloid chitosan (100%) and low activity toward powder chitosan (1.77%) and carboxymethyl cellulose (2.39%) [18]. Also, the CHOE’s substrate specificity with high activity toward colloid chitosan and low activity toward CMC is in accordance with the chitosanase (Csn-PD) from *P. dendritiformis* [4]. It is demonstrated that CHOE has a high specific activity toward the colloidal chitosan substrate, which suggests its potential use for industrial application.

**Table 2:** Substrate specificity of CHOE

Substrate	Relative activity (%)
Colloidal chitosan	100 ± 2.11
Chitosan powder	3.34 ± 0.21
Carboxymethyl chitosan	4.73 ± 0.46
Sodium carboxymethylcellulose	8.21 ± 0.72

The activity for hydrolyzing colloidal chitosan was taken as 100%

#### Kinetics of CHOE

**Fig. 8:**
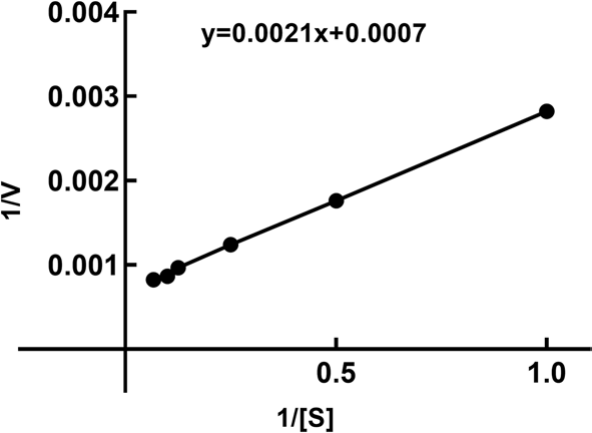
Lineweaver-Burk double reciprocal plot of CHOE

With the colloidal chitosan as the substrate at the concentrations of 0.05–1.5% in the sodium acetate of pH 5.5 at 65 °C, the kinetic parameters of CHOE were determined. *V*_max_ and *K*_m_ of the purified CHOE were calculated to be 1401.9 µmol/min/mg and 3.03 mg/ml, respectively, using the Lineweaver-Burk double reciprocal plot (Fig. 8). The *V*_max_ of CHOE was higher than those of various chitosanases, such as SaCsn46A from *S. avermitilis* (562.32 U/min/mg), and Csn21c from *S. albolongus*, GsCsn46A from *G. sunshinyii* (358.65 U/min/mg), and PoCSN75 from *Penicillium oxalicum* M2 (4.36 U/mL) [16, 18, 42, 43]. The *K*_m_ value of CHOE (3.03 mg/mL) was low than that of Csn21c from *S. albolongus* (7.4 mg/mL), while higher than those of GsCsn46A from *G. sunshinyii* (1.97 mg/mL) and the chitosanase from *Bacillus* sp. TS (1.09 mg/mL) [16, 37, 43]. Notably, activities and properties of the chitosanases from the *Bacillus* genus were usually attractive, which emphasizes their importance and superiority in industrial application.

## Conclusion

A chitosanase-producing strain was isolated and identified as *B. cereus* TY24. The chitosanase-encoding gene (*choe*) was cloned and expressed in *E. coli* BL21(DE3). The chitosanase (CHOE) that was discovered from *B. cereus* TY24, belongs to the family GH-8. Amazingly, CHOE showed remarkable acid stability and thermo-stability that are usually concerned for the industrial application, also chitosanase activity of CHOE was remarkably enhanced by various surfactants. This study indicates the potential application of CHOE from *B. cereus* TY24 in utilizing chitosan resources. Further studies will focus on illustrating the thermostability mechanism of CHOE and improving catalytic efficiency using protein engineering.

## Supplementary Information

**Additional file 1:** **Figure S1**. The original picture of Fig. 5a. The expressed chitosanase was verified by SDS-PAGE analysis. Lane M, protein standard molecular weight; lane 1, supernatant of *E. coli* BL21 (DE3)/pET28a(+)-choe cell lysate induced by IPTG; lane 2, supernatant of *E. coli* BL21 (DE3)/pET28a(+) cell lysate as control. **Figure S2**. The original picture of Fig. 5b. The purified recombinant chitosanase was detected by SDS-PAGE. Lane M, protein standard molecular weight; lane 1, the purified recombinant chitosanase.

## Data Availability

The results of the datasets analyzed during the current study were included in the manuscript and the nucleotide sequence of 16 S rDNA of *Bacillus cereus* TY24 was available in the NCBI (the accession number ON506254). Any additional information used and analyzed for the current study is available from the corresponding author upon reasonable request.
