# Supplementary material for: Gene cloning and molecular characterization of a thermostable chitosanase from Bacillus cereus TY24
Source: BMC Biotechnol. 2022 Oct 27;22:30. doi: 10.1186/s12896-022-00762-6 (PMC9615241; doi:10.1186/s12896-022-00762-6)
Supplement: Supplementary file 1 — Additional file 1: Figure S1. The original picture of Fig. 5a. The expressed chitosanase was verified by SDS-PAGE analysis. Lane M, protein standard molecular weight; lane 1, supernatant of E. coli BL21 (DE3)/pET28a(+)-choe cell lysate induced by IPTG; lane 2, supernatant of E. coli BL21 (DE3)/pET28a(+) cell lysate as control. Figure S2. The original picture of Fig. 5b. The purified recombinant chitosanase was detected by SDS-PAGE. Lane M, protein standard molecular weight; lane 1, the purified recombinant chitosanase. [file 12896_2022_762_MOESM1_ESM.pdf]

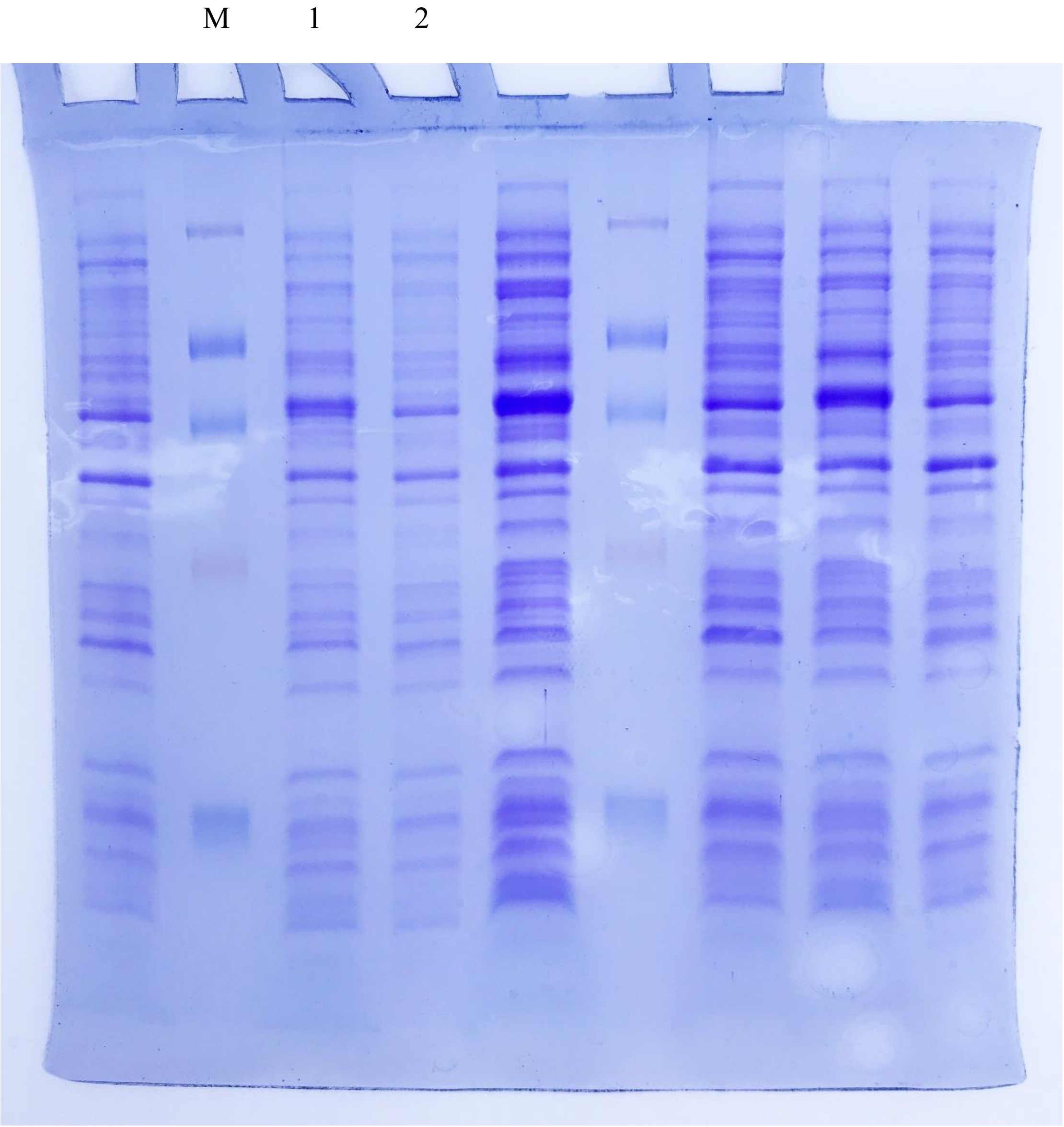

Figure S1. Original electrophoresis gel of Fig. 5a. The expressed chitosanase was verified by SDS-PAGE analysis. Lane M, protein standard molecular weight; lane 1, supernatant of *E. coli* BL21 (DE3)/pET28a(+)-*choe* cell lysate induced by IPTG; lane 2, supernatant of *E. coli* BL21 (DE3)/pET28a(+) cell lysate as control.

M 1

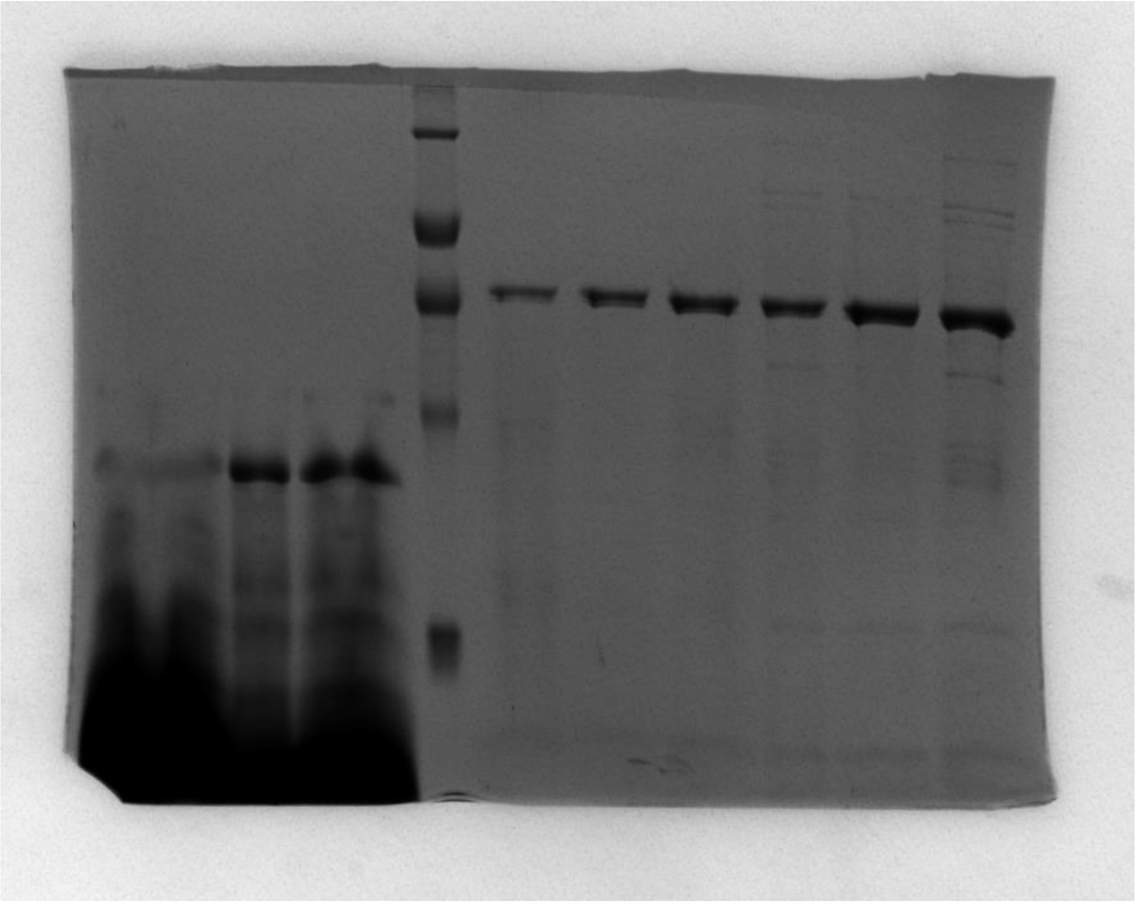

Figure S2. Original electrophoresis gel of Fig. 5b. The purified recombinant chitosanase was detected by SDS-PAGE. Lane M, protein standard molecular weight; lane 1, the purified recombinant chitosanase.
